# Prevalence, patterns and predictors of depression treatment among community-dwelling older adults with stroke in the United States: a cross sectional study

**DOI:** 10.1186/s12888-018-1723-x

**Published:** 2018-05-16

**Authors:** Sandipan Bhattacharjee, Majed Al Yami, Sawsan Kurdi, David Rhys Axon

**Affiliations:** 1 Department of Pharmacy Practice and Science, College of Pharmacy, The University of Arizona, 1295 North Martin Avenue, Tucson, AZ 85721 USA; 2 Health Outcomes & PharmacoEconomic Research (HOPE) Center, College of Pharmacy, The University of Arizona, Tucson, AZ USA

**Keywords:** Stroke, Depression, Antidepressants, Psychotherapy

## Abstract

**Background:**

Depression is one of the most common psychiatric conditions among stroke survivors and is associated with several negative health outcomes. However, little is known about the depression treatment patterns among stroke survivors. The objective of this study was to examine national-level prevalence, patterns and predictors of depression treatment among community-dwelling stroke survivors.

**Methods:**

This study adopted a retrospective, cross-sectional study design using multiple years of Medical Expenditure Panel Survey (MEPS) (2002–2012) data. The study population consisted of older adults (age ≥ 50 years) who (i) were stroke survivors (ICD-9-CM codes of 430–438), (ii) did not die during the calendar year, and (iii) had co-occurring depression (ICD-9-CM code of 296.xx, or 311.xx). Depression treatment, identified by antidepressant medication and/or psychotherapy use, was the dependent variable of this study. Multinomial logistic regression analysis was conducted to examine the association of individual level factors with depression treatment among stroke survivors with co-occurring depression.

**Results:**

The final study sample consisted 370 (unweighted) community-dwelling older adults with self-reported stroke and depression. The prevalence of co-occurring depression among stroke survivors was 22.03% [95% Confidence Interval (CI) 19.7–24.4%]. An overwhelming majority (87.6%) of stroke survivors with co-occurring depression reported some form of depression treatment. Antidepressants only and combination therapy was reported by 74.8% (95% CI, 71.6–78.0%] and 12.8% (95% CI, 10.5–15.1%) by stroke survivors with co-occurring depression respectively. Approximately, 61% of stroke survivors with co-occurring depression reported using SSRIs, followed by SNRIs (15.2%), miscellaneous antidepressants (12.1%), TCAs (9.8%), phenylpiperazine antidepressants (5.2%), and tetracyclic antidepressants (4%). Sertraline (15.8, 95% CI, 12.7–19.0%) had the highest reported use among individual antidepressants.

**Conclusions:**

Vast majority (nearly 90%) of the study sample received some form of depression treatment and several individual level factors (such as age, education) were associated with the report of depression treatment use. Future longitudinal studies are warranted to assess the comparative treatment benefits of antidepressants, psychotherapy and their combination. Healthcare providers should carefully assess the risks and benefits of antidepressant (such as SSRIs or TCAs) use in this vulnerable population prior to their use.

**Electronic supplementary material:**

The online version of this article (10.1186/s12888-018-1723-x) contains supplementary material, which is available to authorized users.

## Background

Stroke is a common and costly disease, which is a major burden on patients’ lives and the health system economy. On average, every 40 s, someone in the United States (US) has either a new or recurrent stroke, with an annual incidence of 795,000 [1]. Other than being the fifth leading cause of mortality, stroke is the leading cause of long-term adult disability in the US [1]. Due to advances in awareness, risk factor control, and the aging population, stroke survivors’ prevalence has been increasing [1]. However, recovery after stroke takes time, and usually stroke survivors live with some degree of functional disability and depression is a common sequelae after stroke [2]. A pooled estimate indicates that depressive symptoms are present in one third of all stroke survivors at any time during the follow-up [3]. Another study reported that pooled prevalence of minor and major depression in community based setting after stroke are 9 and 14%, respectively [4].

Co-occurring depression among stroke survivors is associated with poor rehabilitation outcome [5], compromised quality of life [6], social isolation, poor medications adherence [7], and higher rate of mortality [8]. Given the array of negative outcomes associated with co-occurring depression among stroke survivors, it is critical to treat depression in this vulnerable population, which in turn may improve patients’ health outcomes and quality of life. In general, depression can be treated by using antidepressants, psychotherapy, or both. Existing randomized controlled trials (RCTs) have demonstrated the benefits of these depression treatment modalities in reducing depression burden among stroke survivors [9–14]. Moreover, in a Cochrane review of 12 clinical trials, the use of antidepressant treatment in older adults(age range of 60–78), was associated with an improvement in rates of depression (OR 0.47, 95% CI 0.22–0.98) [15]. A recent systematic review and meta-analysis of RCTs investigating the effectiveness of antidepressants in post-stroke depression found a standardized mean effect size of − 0.96 (95% CI = − 1.41, − 0.51) between antidepressants versus placebo, indicating a significant advantage of antidepressants over placebo (*p* < 0.0001) [16]. Furthermore, Mitchell et al. found that a combination of antidepressant and behavioral therapy was more effective in reducing depression compared to antidepressants alone [11].

Despite the evidence of the efficacy of depression treatment modalities (antidepressants only, psychotherapy only, and antidepressant and psychotherapy combination),, to the best of our knowledge, no study to date has been conducted to examine the national-level prevalence, patterns and predictors of depression treatment among community-dwelling stroke survivors with co-occurring depression in the United States (US). Examining the current treatment patterns can help to understand whether the demonstration of depression treatment efficaciousness among stroke survivors from RCTs have helped in adoption of depression treatment into real-world clinical practice. Moreover, determining the predictors of depression treatment among stroke survivors can provide insights into factors where there is/are need of intervention(s) to improve depression care. Hence, this study is a first of its kind to examine national-level prevalence, patterns and predictors of depression treatment among community-dwelling stroke survivors in the US.

## Methods

### Study design

We adopted a retrospective, cross-sectional study design using pooled data from Medical Expenditure Panel Survey (MEPS) (2002–2012). Respondents of the MEPS are followed for two years. In order to eliminate the chances of including the same respondent between consecutive years, we used alternate years of data (2002, 2004, 2006, 2008, 2010, and 2012) [17]. It is recommended that multiple years of MEPS data be used to achieve adequate sample size [18].

### Data source

MEPS is a nationwide survey of US citizens, their healthcare providers and employers which records healthcare cost, use and insurance coverage [19], collected by the Agency for Healthcare Research and Quality (AHRQ). MEPS uses the sampling framework of the National Health Interview Survey (NHIS) and contains information about medical and mental health conditions, as well as services and treatments such as prescription drugs and counseling therapies. The survey oversamples minority groups and disabled people in order to achieve nationally representative estimates. The survey consists of a household component (MEPS-HC) and an insurance component (MEPS-IC). The MEPS-HC uses a nationally representative sample panel of households selected annually whereby household respondents provide information about their medical conditions. This information is then converted into International Classification of Diseases, 9th Edition, Clinical Modification (ICD-9-CM) codes and clinical classification codes by professional coders. Data in each panel are collected for two calendar years. Information from MEPS-HC was used to obtain data of self-reported medical conditions and socio-demographic data. The MEPS-IC is conducted by the U.S. Census Bureau and contains information on establishment characteristics, whether an establishment offers health insurance, and details on health plans. Household respondents medical conditions have been found to typically correlate with medical care provider data. It has been demonstrated that MEPS participant reported conditions are consistent with those provided by their medical providers for chronic conditions (such as diabetes, mental health, hypertension) with a median sensitivity of 70% [20]. The MEPS Medical Conditions file was used to identify the comorbidities, the Prescription Use file was used to identify the medication use, and Outpatient and Office-based Provider Visits files were used to identify psychotherapy use [19].

### Study population

The study population consisted of older adults (age ≥ 50 years) who (i) were stroke survivors, (ii) did not die during the calendar year, and (iii) had co-occurring depression. Stroke was identified by using International Classification of Diseases, Ninth Revision, Clinical Modification (ICD-9-CM) code of 430.xx-438.xx [21]. These ICD-9-CM codes are used by the American Heart Association to report stroke statistics [22] and have also shown high sensitivity (≥ 82%), specificity (≥ 95%), and positive predictive value (≥ 81%) to identify overall diagnosis of acute or preexisting stroke [23]. Co-occurring depression was identified by using ICD-9-CM code of 296.xx, or 311.xx [24]. As MEPS publically available medical conditions file provides only three digit ICD-9-CM codes, there is a possibility of including some individuals with bipolar disorder with the use of ICD-9-CM code of 296.xx. However, it has been observed that most of the individuals with an ICD-9-CM code of 296.xx have major depressive disorder and overwhelming majority (> 90%) of them have an ICD-9-CM code of 311 that corresponds to unspecified depression [25]. Moreover, existing studies using MEPS data have used ICD-9-CM codes of 296.xx and 311.xx to identify depression [26, 27].

### Dependent variable

Depression treatment constituted the dependent variable. Depression treatment was identified by antidepressant medication and/or psychotherapy use. Antidepressant use was ascertained from the prescription drug files. Multum Lexicon therapeutic class code (second level) of “249” was used to identify antidepressant use. The third level of Multum Lexicon therapeutic class codes were used to identify the subclasses of antidepressants. Antidepressants were classified into the following categories: (i) Selective serotonin reuptake inhibitors (SSRIs); (ii) Serotonin–norepinephrine reuptake inhibitors (SNRIs); (iii) Tricyclic antidepressants (TCAs); (iv) Monoamine oxidase inhibitor (MAO inhibitors); (v) phenylpiperazine antidepressants; (vi) Tetracyclic antidepressants; and (vii) Miscellaneous antidepressants. An additional file is provided showing detailed Multum Lexicon codes used to identify these subclasses of antidepressants [see Additional file 1: Table S1]. National Drug Codes (NDCs) were used to identify individual antidepressants. Psychotherapy use was obtained from the provider visits of outpatient and office-based files. Stroke survivors with co-occurring depression, who reported at least one visit for psychotherapy or mental health counseling services, were included in the group of psychotherapy receipt [28].

We defined depression treatment in two different ways. In the first measure, depression treatment was categorized as (i) receipt of antidepressants and/or psychotherapy; and (ii) no depression treatment. For the second measure, the depression treatment was classified into four groups: (i) antidepressant use alone; (ii) psychotherapy alone; (iii) antidepressant with psychotherapy; and (iv) no depression treatment. As the sample size of stroke survivors with co-occurring depression, who used psychotherapy alone (Unweighted *N* = 13) was very small, they were not included in the final study sample.

### Independent variables

This study was conceptualized using Ronal M. Andersen’s Behavioral Model of Health Services Use framework to investigate the association between predisposing, enabling, need, external environmental, and personal health practices factors with depression treatment [29]. The predisposing factors included age (50–64 years, 65 years and older), gender (male; female), and race/ethnicity (white and others). Enabling factors included education (less than high school; up to high school; and higher than high school), marital status (married and others), type of insurance (public; private; and uninsured), and poverty status [poor or near poor (< 200% of federal poverty level); middle income or high income (≥ 200% of federal poverty level)]. Need factors included chronic diseases (e.g. arthritis, osteoporosis, diabetes, thyroid disorder, heart disease, hypertension, asthma, gastro esophageal reflux disorder, depression, anxiety and other neurological conditions), limitations of activities of daily living (ADLs) (yes/no), limitations of instrumental activities of daily living (IADLs) (yes/no), activities limitations in work or housework (yes/no), functional activities limitations (yes/no), perceived physical health status (excellent/very good; good; and fair/poor), perceived mental health status (excellent/very good; good; and fair/poor), and pain (quite or extreme pain; moderate or little pain; and no pain). If a survey respondent required help or supervision on any of the ADLs (eating, dressing, bathing, toileting, getting in and out of bed, and mobility inside own residence) for at least another three months from the date of data collection (during any of the multiple interviews in a year), then they were considered to have ADL limitations. IADL limitations were defined similarly based on whether they required help or supervision with using the telephone, paying bills, taking medications, preparing light meals, doing laundry, or going shopping. MEPS participants were considered to have functional limitations if they had difficulties performing specific physical actions, or had problems with cognition, vision and hearing. The series of questions asked to evaluate difficulties with physical actions for at least three more months were: difficulty lifting 10 pounds, difficulty walking up 10 steps, difficulty walking 3 blocks, difficulty walking a mile, difficulty standing 20 min, difficulty bending or stooping, difficulty reaching over head, difficulty using fingers to grasp. Cognitive limitations were identified with a “yes” response to any of the following questions: (i) experienced confusion or memory loss, (ii) had problems making decisions, or (iii) required supervision for their own safety. Vision or hearing problems were identified by asking the respondent whether they had difficulty seeing or hearing respectively. Activities limitation was identified based on whether MEPS participants had any limitation in work, housework, or school. Perceived physical and mental health status were collected during any of the interview rounds by asking MEPS respondents to rate their physical and mental health status into any of the following categories: excellent, very good, good, fair, and poor. Personal health practices consisted of body mass index (BMI) categories (under or normal; and overweight or obese), and smoking status (current smoker; and others). External environmental factors consisted of region (Mid-West; Northeast; West; and South), and metropolitan status (metropolitan; and non-metropolitan).

### Pain

Antidepressants are prescribed at certain times to alleviate pain associated with different chronic conditions [30] and hence can influence depression treatment patterns among stroke survivors with co-occurring depression. In order to minimize the effect of pain on depression treatment among stroke survivors with co-occurring depression, we adjusted pain as one of the variables in the multivariate regression models. MEPS respondents were asked if - “*During past 4 weeks, pain interfered with normal work outside the home and housework*”, which was used to construct the pain variable [31]. The pain variable was self-reported by MEPS respondents on a 5-point scale: (i) not at all; (ii) a little bit; (iii) moderately; (iv) quite a bit; and (v) extremely. We categorized pain into three categories for this study: (i) quite a bit/extreme pain; (ii) a little bit or moderate pain; and (iii) no pain.

### Statistical analysis

Univariate analyses were conducted using chi-square tests to ascertain the statistically significant differences among different depression treatment groups (antidepressant use alone, antidepressant and psychotherapy combination and no depression treatment) in the study population. Multinomial logistic regression was conducted to assess the factors (predisposing, enabling, need, personal health choices, and external environmental factors) associated with depression treatment among stroke survivors with co-occurring depression. The levels of the depression treatment (dependent variable) consisted of: (i) antidepressant use alone; (ii) antidepressant and psychotherapy (combination therapy); and (iii) no depression treatment (which served as the reference group for the multinomial logistic regression). Sensitivity analyses was conducted by using logistic regression with any form of depression treatment (antidepressant and/or psychotherapy) compared to no depression treatment as dependent variable adjusting for predisposing, enabling, need, personal health practices, and external environmental factors. National estimates were obtained by adjusting for the complex survey design of MEPS. SAS version 9.4 (SAS institute Inc., Cary, NC, USA) was used to carry out all the analyses.

## Results

The final study sample consisted 370 (unweighted) community-dwelling older adults with self-reported stroke and depression who were alive during the calendar year (2002, 2004, 2006, 2008, 2010, and 2012). The prevalence of co-occurring depression among stroke survivors was 22.03% [95% Confidence Interval (CI) 19.7–24.4%]. An overwhelming majority (87.6%) of stroke survivors with co-occurring depression reported some form of depression treatment. Antidepressants only and combination therapy was reported by 74.8% (95% CI, 71.6–78.0%] and 12.8% (95% CI, 10.5–15.1%) respectively by stroke survivors with co-occurring depression. Only 12.4% (95% CI, 9.9–14.8%) of stroke survivors with co-occurring depression reported that they did not receive any form of depression treatment.

Table 1 shows the overall distribution of predisposing, enabling, need, personal health practices and external environmental factors among stroke survivors with co-occurring depression as well as the distribution of these factors in different depression treatment groups. Overall, majority of stroke survivors with co-occurring depression were 65 years and older (60.4%), females (62.4%), whites (77.3%), reported fair or poor perceived physical health status (62.5%), experienced some form of pain (87.9%), reported functional disability (89.8%), had some chronic diseases (66.2%), and resided in metropolitan area (78%). From Table 1, it can be observed that there were subgroup differences in terms of age, race/ethnicity, education, poverty status, insurance, perceived mental health status, pain, ADL disability, chronic disease group, BMI status and region. For example, a greater proportion (64.9%) of stroke survivors with co-occurring depression who received combination therapy were in the age range of 50–64 years, whereas the majority (65.2%) of stroke survivors with co-occurring depression receiving antidepressants only were 65 years or older.

**Table 1:** Descriptive Statistics of Stroke Survivors with Co-occurring Depression Medical Expenditure Panel Survey (2002–2012)

	All (*N* = 370)	No Dep Tx (*N* = 55)	AD only (*N* = 268)	AD & Psych (*N* = 47)	Sig
	Wt.%	Wt.%	Wt.%	Wt.%	
		12.4	74.8	12.8	
Predisposing Factors					
Age					
50–64 years	39.6	42.8	34.8	64.9	***
65,+ years	60.4	57.2	65.2	35.1	
Gender					
Female	62.4	65.3	62.1	61.9	
Men	37.6	34.7	37.9	38.1	
Race/Ethnicity					
White	77.3	67.4	78.1	82.3	*
Other	22.7	32.6	21.9	17.7	
Enabling Factors					
Marital Status					
Married	49.4	41.5	51.4	45.2	
Other	50.6	58.5	48.6	54.8	
Education					
LT HS	25.7	39.7	24.5	17.3	***
HS	33.2	32.6	31.0	47.6	
> HS	41.0	27.7	44.5	35.1	
Poverty Status					
Poor	15.9	26.2	14.0	17.6	**
Near Poor	24.3	32.0	23.4	22.2	
Middle Income	33.2	29.9	33.8	33.0	
High Income	26.6	11.9	28.9	27.2	
Insurance					
Public	47.2	59.3	42.8	61.1	***
Others	52.8	40.7	57.2	38.9	
Need Factors					
Perceived Physical Health Status					
Ex/vgood	12.4	12.0	13.2	8.8	
Good	25.1	28.5	23.1	33.6	
Fair/poor	62.5	59.5	63.8	57.6	
Perceived Mental Health Status					
Ex/vgood	22.9	36.1	23.0	9.2	*
Good	38.0	28.2	39.2	39.8	
Fair/poor	39.2	35.7	37.7	50.9	
Pain					
Quite/Extreme	46.4	51.7	44.3	53.6	***
Little/Moderate	41.5	28.4	45.4	31.3	
No Pain	12.1	19.8	10.3	15.0	
ADL Disability					
Yes	31.2	32.9	33.1	18.3	*
No	68.8	67.1	66.9	81.7	
IADL Disability					
Yes	51.7	47.0	52.9	49.6	
No	48.3	53.0	47.1	50.4	
Functional Disability					
Yes	89.8	94.4	89.7	85.9	
No	10.2	5.6	10.3	14.1	
Activities Disability					
Yes	80.2	75.7	80.6	82.4	
No	19.8	24.3	19.4	17.6	
Chronic Disease Groups					
None	33.8	26.0	34.8	35.8	**
One	30.2	32.5	29.3	33.3	
Two	24.5	20.8	26.8	14.7	
Three or more	11.4	20.7	9.0	16.3	
Personal Health Practices Factors					
BMI Status					
Und/normal	58.8	77.5	58.4	43.6	***
Overwt/Obese	41.2	22.5	41.6	56.4	
Smoking Status					
Current smoker	16.3	8.1	17.4	17.6	
Other	83.7	91.9	82.6	82.4	
External Environmental Factors					
Area of Residence					
Metropolitan	78.0	84.9	78.2	70.3	
Non-Metropolitan	22.0	15.1	21.8	29.7	
Region					
Northeast	18.9	19.9	15.5	37.6	***
Mid-west	21.9	8.5	25.0	16.4	
South	37.7	48.1	38.8	21.8	
West	21.5	23.5	20.7	24.2	

Based on 370 older adults (age ≥ 50 years) with self-reported stroke and depression who were alive during the calendar year (2002, 2004, 2006, 2008, 2010, and 2012)

*Abbreviations*- *No Dep Tx* No Depression Treatment, *AD only* Antidepressants only, *AD & Psych* Antidepressants with Psychotherapy, *LT HS* Less than High School, *HS* High School, *Wt%* Weighted percentage, *Sig* Significant difference, *Ex/vgood* Excellent or Very Good, *ADL* Activity of Daily Living, *IADL* Instrumental Activity of Daily Living, *Und/normal* Underweight or Normal, *Overwt/Obese* Overweight or Obese, *BMI* Body Mass Index

Asterisks represent statistical significance between the different depression treatment groups [No Depression Treatment (Unweighted N = 55); Antidepressants only (Unweighted N = 268); and Antidepressants with Psychotherapy (Unweighted N = 47)] based on chi-square tests

***p < 0.001; ** 0.001 ≤ p < 0.01; *0.01 ≤ p < 0.05

Table 2 provides the nationally representative patterns of depression treatment among stroke survivors with co-occurring depression. Approximately, 61% of stroke survivors with co-occurring depression reported using SSRIs, followed by SNRIs (15.2%), miscellaneous antidepressants (12.1%), TCAs (9.8%), phenylpiperazine antidepressants (5.2%), and tetracyclic antidepressants (4%). Sertraline (15.8, 95% CI, 12.7–19.0%) had the highest reported use among individual antidepressants (data not presented in table). Other individual antidepressant use in the study population that should be noted include citalopram (13.9, 95% CI, 11.1–16.7%), paroxetine (12.5, 95% CI, 9.9–15.1%), escitalopram (11.4, 95% CI, 8.1% - 14.6), duloxetine (8.9, 95% CI, 6.2–11.6%), fluoxetine (8.8, 95% CI, 6.8–10.8%), bupropion (8.6, 95% CI, 7.2–10.1%), venlafaxine (7.1, 95% CI, 4.3–9.9%), trazodone (6.6, 95% CI, 4.8–8.4%), amitriptyline (5.5, 95% CI, 3.5–7.5%) and mirtazapine (4.7, 95% CI, 1.9% - 7.4).

**Table 2:** Dep Tx Patterns among Stroke Survivors with Co-occurring Depression Medical Expenditure Panel Survey (2002–2012)

	Wt.%
Any form of Dep Tx	87.6
SSRI	60.9
SNRI	15.2
TCA	9.8
PPAZ	5.2
Tetra Cyc	4.0
Misc ADP	12.1

Based on 370 older adults (age ≥ 50 years) with self-reported stroke and depression who were alive during the calendar year (2002, 2004, 2006, 2008, 2010, and 2012)

*Abbreviations* - *Dep Tx* Depression Treatment, *SSRI* Selective Serotonin Reuptake Inhibitor, *SNRI* Serotonin–Norepinephrine Reuptake Inhibitor, *TCA* Tricyclic Antidepressants, *PPAZ* Phenylpiperazine Antidepressants, *Tetra Cyc* Tetracyclic Antidepressants, *Misc ADP* Miscellaneous Antidepressants, *Wt%* Weighted percentage (nationally representative)

Total number of individual antidepressant classes may not add up to 100% due to intra-class polypharmacy

Denominator for Wt.% calculations for individual types of antidepressant classes was the total analytic sample (Unweighted N = 370)

Table 3 summarizes the findings from the multinomial logistic regression analysis. Several individual-level factors were associated with depression treatment. For example, among stroke survivors with co-occurring depression, those who were 65 years and older were nearly six times more likely [Adjusted Odds Ratio (AOR): 5.80, 95% CI 2.48–13.5] to report use of antidepressants only compared to those who were 50–64 years old. Stroke survivors with co-occurring depression who had less than high school education were 92% (AOR: 0.08, 95% CI 0.02–0.37) less likely to report the use combination therapy compared to those with higher than high school education. Details of the multinomial logistic regression are presented in Table 3 Sensitivity analyses with depression treatment (yes/no) showed similar findings (Table 4).

**Table 3:** Multinomial Logistic Regression among Stroke Survivors with Co-occurring Depression in terms of Depression Treatment Medical Expenditure Panel Survey (2002–2012)

	AD Only (*N* = 268)			AD & Psych (*N* = 47)		
	AOR	95% CI	Sig	AOR	95% CI	Sig
Predisposing Factors						
Age						
65,+ yrs. vs 50–64 yrs	5.80	[2.48,13.5]	***	1.16	[0.42,3.18]	
Gender						
Female vs Men	0.69	[0.33,1.42]		0.53	[0.21,1.31]	
Race/Ethnicity						
Other vs White	1.60	[0.77,3.32]		1.45	[0.58,3.61]	
Enabling Factors						
Marital Status						
Other vs Married	1.02	[0.45,2.31]		1.17	[0.32,4.27]	
Education						
LT HS vs > HS	0.07	[0.02,0.27]	***	0.08	[0.02,0.37]	**
HS vs > HS	0.24	[0.08,0.75]	*	0.57	[0.13,2.53]	
Insurance						
Public vs others	0.75	[0.22,2.54]		3.54	[0.65,19.2]	
Poverty Status						
Mid Inc./High Inc. vs Poor/Near Poor	1.60	[0.66,3.87]		3.90	[1.19,12.8]	*
Need Factors						
Pain						
Quite/Extreme vs No pain	1.09	[0.32,3.67]		1.02	[0.22,4.68]	
Little/Moderate vs No pain	1.97	[0.54,7.20]		1.11	[0.23,5.33]	
Perceived Physical Health Status						
Good vs Ex/vgood	0.49	[0.08,2.93]		0.72	[0.09,5.82]	
Fair/poor vs Ex/vgood	0.72	[0.22,2.41]		0.19	[0.06,0.60]	**
Perceived Mental Health Status						
Good vs MH Ex/vgood	4.55	[2.26,9.15]	***	11.04	[3.33,36.6]	***
Fair/poor vs MH Ex/vgood	3.60	[1.40,9.27]	**	19.19	[5.41,68.1]	***
ADL Disability						
Yes vs No	2.05	[0.72,5.86]		0.39	[0.11,1.34]	
IADL Disability						
Yes vs No	1.57	[0.52,4.78]		2.20	[0.64,7.50]	
Functional Disability						
Yes vs No	0.19	[0.04,0.83]	*	0.31	[0.06,1.65]	
Activities Disability						
Yes vs No	3.63	[0.89,14.8]		3.73	[0.71,19.6]	
Chronic Disease Groups						
One vs None	0.54	[0.17,1.69]		1.55	[0.41,5.86]	
Two vs None	0.91	[0.27,3.06]		1.33	[0.36,4.89]	
Three or more vs None	0.19	[0.07,0.48]	***	0.62	[0.06,5.92]	
CVD						
Yes vs No	0.02	[0.00,1.34]		0.08	[0.00,4.72]	
Anxiety						
Yes vs No	0.79	[0.31,1.99]		1.09	[0.40,2.93]	
Hyperlipidemia						
Yes vs No	1.35	[0.69,2.66]		2.73	[0.94,7.96]	
Personal Health Practices Factors						
BMI Status						
Overwt/Obese vs Und/normal	5.18	[1.93,13.8]	**	9.35	[2.98,29.3]	***
Smoking Status						
Curr smoker vs Other	6.40	[1.51,27.0]	*	6.04	[1.42,25.6]	*
External Environmental Factors						
Metropolitan Status						
Non-Metropolitan vs Metropolitan	1.53	[0.67,3.46]		3.42	[1.13,10.2]	*
Region						
Mid-west vs Northeast	4.05	[1.28,12.7]	*	0.64	[0.17,2.40]	
South vs Northeast	0.83	[0.30,2.27]		0.20	[0.06,0.63]	**
West vs Northeast	0.53	[0.12,2.37]		0.36	[0.05,2.51]	

Based on 370 older adults (age ≥ 50 years) with self-reported stroke and depression who were alive during the calendar year (2002, 2004, 2006, 2008, 2010, and 2012)

*Abbreviations* -*No Dep Tx* No Depression Treatment, *AD only* Antidepressants only, *AD & Psych* Antidepressants with Psychotherapy, *LT HS* Less than High School, *HS* High School, *Wt%* Weighted percentage, *Sig* Significant difference, *Ex/vgood* Excellent or Very Good, *ADL* Activity of Daily Living, *IADL* Instrumental Activity of Daily Living, *Und/normal* Underweight or Normal, *Overwt/Obese* Overweight or Obese, *BMI* Body Mass Index, *AOR* Adjusted odds ratio, *Mid Inc./High Inc.* Middle Income or High Income

Asterisks represent statistical significance group differences by type of treatment compared to the reference group based on multinomial logistic regression. The reference group for the dependent variable in the multinomial logistic regression was “No Depression Treatment” (*N* = 55)

***p < 0.001; ** 0.001 ≤ p < 0.01; *0.01 ≤ p < 0.05

**Table 4:** Logistic Regression among Stroke Survivors with Co-occurring Depression in terms of Depression Treatment Medical Expenditure Panel Survey (2002–2012)

	AOR	95% CI	Sig
Predisposing Factors			
Age			
65,+ yrs. vs 50–64 yrs	4.65	[2.10,10.2]	***
Gender			
Female vs Men	0.71	[0.36,1.41]	
Race + Ethnicity			
Other vs White	1.54	[0.78,3.05]	
Enabling Factors			
Marital Status			
Other vs Married	1.02	[0.46,2.26]	
Education			
LT HS vs > HS	0.08	[0.02,0.26]	***
HS vs > HS	0.29	[0.10,0.80]	*
Insurance			
Public vs others	0.89	[0.28,2.81]	
Poverty Status			
Mid Inc./High Inc. vs Poor/Near Poor	1.87	[0.87,4.01]	
Need Factors			
Pain			
Quite/Extreme vs No pain	1.20	[0.39,3.67]	
Little/Moderate vs No pain	1.99	[0.60,6.65]	
Perceived Physical Health Status			
Good vs Ex/vgood	0.56	[0.10,3.02]	
Fair/poor vs Ex/vgood	0.65	[0.21,2.01]	
Perceived Mental Health Status			
Good vs Ex/vgood	4.55	[2.37,8.72]	***
Fair/poor vs Ex/vgood	4.05	[1.69,9.73]	**
ADL Disability			
Yes vs No	1.68	[0.64,4.37]	
IADL Disability			
Yes vs No	1.62	[0.58,4.49]	
Functional Disability			
Yes vs No	0.20	[0.05,0.84]	*
Activities Disability			
Yes vs No	3.60	[0.97,13.2]	
Chronic Disease Groups			
One vs None	0.64	[0.22,1.86]	
Two vs None	1.00	[0.34,2.98]	
Three or more vs None	0.21	[0.08,0.52]	***
CVD			
Yes vs No	0.02	[0.00,1.12]	
Anxiety			
Yes vs No	0.88	[0.38,2.01]	
Hyperlipidemia			
Yes vs No	1.55	[0.82,2.93]	
Personal Health Practices Factors			
BMI Status			
Overwt/Obese vs Und/normal	5.71	[2.27,14.3]	***
Smoking Status			
Curr smoker vs Other	6.30	[1.65,240]	**
External Environmental Factors			
Metropolitan Status			
Non-Metropolitan vs Metropolitan	1.64	[0.77,3.49]	
Region			
Mid-west vs Northeast	2.80	[0.86,9.14]	
South vs Northeast	0.60	[0.22,1.66]	
West vs Northeast	0.41	[0.10,1.78]	

Based on 370 older adults (age ≥ 50 years) with self-reported stroke and depression who were alive during the calendar year (2002, 2004, 2006, 2008, 2010, and 2012)

*Abbreviations*- *yrs.* years, *LT HS* Less than High School, *HS* High School, *Wt%* Weighted percentage, *Sig* Significant difference, *Ex/vgood* Excellent or Very Good, *ADL* Activity of Daily Living, *IADL* Instrumental Activity of Daily Living, *Und/normal* Underweight or Normal, *Overwt/Obese* Overweight or Obese, *BMI* Body Mass Index, *AOR* Adjusted odds ratio, *Mid Inc./High Inc.* Middle Income or High Income

Asterisks represent statistical significance group differences by type of treatment (any form of depression treatment and no depression treatment) compared to the reference group based on binomial logistic regression. The reference group for the dependent variable in the binomial logistic regression was “No Depression Treatment”

***p < 0.001; ** 0.001 ≤ p < 0.01; *0.01 ≤ p < 0.05

## Discussion

The current study estimated the national level prevalence of co-occurring depression among community-dwelling older adults with stroke in the US to be 22.03%. To the best of our knowledge, this is the first study to date to estimate the national-level prevalence of co-occurring depression among community-dwelling stroke survivors in the US. Data from existing studies showed that the prevalence of depression among individuals with stroke varied widely across the world based on the region and method used, ranging from 11 to 79% [32–43], with epidemiological studies showing the co-occurrence of depression in 30% of stroke cases [44]. A recent study by McCarthy et al. (2016) using data from the greater Cincinnati/Northern Kentucky stroke study in the US found that 37% of stoke survivors were at risk for depression at 3-months post-stroke [45]. Another recent study by Fei et al. (2016) using data from the Prevent Recurrent All-Inner city Stroke through Education (PRAISE) study of 556 individuals in New York City in the US found that 31% of stroke survivors had post stroke depression [46]. Prevalence of depression observed in our study was slightly lower compared to the available estimates from the recent US studies [45, 46], that can be explained by the fact that these are self-reported by MEPS respondents, which are often associated with stigma of reporting mental illnesses, as well as the possibility of under-diagnosis of depression. Another possible explanation can be that some stroke survivors may not recently report depressive symptoms as they may have been successfully treated and are currently in remission. However, depression can be episodic, so regular depression screening is recommended.

The current study also provides the first national-level estimate of the receipt of depression treatment among community-dwelling stroke survivors with co-occurring depression. Among individual antidepressant classes, the current study found that SSRIs were reported as the mostly used antidepressants, which is not surprising as SSRIs have been found to be effective among stroke survivors in reducing dependency, disability, neurological impairment, anxiety as well as depression [47]. Moreover, in a population-based nested case control study, SSRIs were not found to be associated with recurrent stroke risk [48]. However, this study [48] found that TCAs, were associated with 41% higher likelihood of stroke recurrence. According to the current study findings, TCAs were reported to be used by approximately 10% of the study sample, and given the risk of recurrent stroke, close monitoring should be in place for individuals on TCAs. Another advantage of using SSRIs among stroke survivors with depression is the potential benefit of decreasing platelet aggregation and adhesion. However, this benefit has been examined and observed among individuals with different conditions such as myocardial infarction, depression, and ischemic heart disease (with depression) but not among stroke survivors [49–51]. It should however be kept in mind that SSRIs do have significant side effects as demonstrated by a study among post-menopausal women using the Women’s Health Initiative (WHI) data [52]. This study using the WHI data observed that even though the absolute event risks are low, SSRIs are associated with higher risk of death, and hemorrhagic and fatal stroke [52]. Hence, the benefits to risk profile should always be assessed before using these medications. It is noteworthy to mention that currently there are three ongoing trials (FOCUS, AFFINITY, and EFFECTS) examining the effect of fluoxetine 20 mg daily for six months on different outcomes (such as modified Rankin scale, diagnosis of depression and adherence to medication) among stroke survivors [53]. Furthermore, there is an ongoing real-world study funded by the Patient Centered Outcomes Research Institute (PCORI) assessing the effectiveness of antidepressants among stroke survivors [54]. Findings from these studies have the potential to provide further evidence of antidepressant safety and effectiveness among stroke survivors.

Sertraline was the most frequently used individual antidepressant in this study sample. An open-label study [55] among stroke survivors with depression found that symptoms of depression and anxiety had consistent decline along with improvement in cognitive and functional performances with sertraline treatment and hence findings from this study are unsurprising. Approximately 14% of the current study sample reported using citalopram, which can be explained in the light of fewer side effects observed with citalopram in stroke survivors with depression [56], as well as the chances of significantly improving dexterity [57] after stroke. However, use of some individual antidepressants such as trazodone, mirtazapine and venlafaxine warrants constant monitoring as they have been observed to be associated high risks of several adverse events [58]. This study observed psychotherapy with antidepressant use in only 12.8% of stroke survivors with co-occurring depression. Even though specific data of the prevalence of psychotherapy use among stroke survivors is not available, but an existing study among depressed Medicare beneficiaries during 1992–1999 found psychotherapy use among a quarter of these patients [59]. However, the lower use of psychotherapy observed in the current study can be explained by the decline of outpatient psychotherapy in general population [60] as well as concerns about psychotherapy effectiveness among stroke survivors [61]. Selection of pharmacologic versus non-pharmacologic treatment modality is complicated and depends on several factors. For example, the choice of antidepressant treatment is generally influenced by the presence of multimorbidity, polypharmacy, and predisposition to antidepressant adverse effects [62]. In this context, non-pharmacologic treatment options (such as cognitive behavioral therapy) may present a favorable alternative. However, non-pharmacologic interventions are expensive, time consuming and usually show delayed response [62]. Thus, from a healthcare practice and policy perspective, it may be prudent to carefully weigh these different factors along with patient preferences, which has the potential to improve adherence and outcomes associated with these depression treatment modalities.

In certain cases, no depression treatment option is adopted among stroke survivors with co-occurring depression. In our study, not reporting any form of depression treatment among stroke survivors with co-occurring depression can be due to several reasons such as patient and/or physician preference, suboptimal detection of depression, depression therapy discontinuation due to adverse effects, non-adherence to depression therapy, or the possibility of improvement or remission of depressive symptoms. Future studies are warranted to understand the factors associated with no depression treatment in this vulnerable population.

In terms of predisposing factors, the current study showed that older adults (age ≥ 65 years) in the study sample were more likely to receive antidepressant therapy compared to those within 50–64 years of age. This finding can be partially explained by the overall increase in antidepressant use among older adults in the US from 3% in 1988–1994 [63] to 13.7% during 2007–2010 [64]. Moreover, a study of veterans who survived stroke with an average age of 69.4 years found it was clinically beneficial when patients were dispensed an antidepressant medication [65]. However, caution should be taken with the use of antidepressants in older adults, as with increasing age the chances of developing serotonin syndrome, falls and fractures risk, and mortality increases [58].

With respect to enabling factors, it was observed that stroke survivors with co-occurring depression with lower levels of education had less likelihood of receiving antidepressant therapy or combination therapy. This may be because lower levels of education are consistently associated with limited health literacy [66] and this may lead to less preventative care seeking behavior [67]. Appropriate interventions are required to improve health literacy as individuals with low literacy usually experience 1.5 to 3 times higher chances of poor health outcomes [68]. The current study also observed that stroke survivors with co-occurring depression who belonged to high or middle income status were more likely to receive combination therapy compared to individuals with lower incomes. This finding is not surprising as existing literature suggests that adults from low-income households have several barriers (lack of transport, childcare, health insurance, and difficult working hours) to accessing mental health services such as psychotherapy [69].

In terms of need factors, good and fair/poor perceived mental health status was associated with increased likelihood of antidepressant and combination therapy use. This study finding is consistent with an existing study by Olfson et al. (2006) [70], where longer-term antidepressant use was observed to be associated with perceived fair or poor mental health status, which can be partially attributed to greater motivation from these individuals or their caregivers. In the current study it was observed that having functional disability was associated with lower likelihood of antidepressant use. This finding is counterintuitive as existing double-blind randomized study demonstrated that antidepressant treatment was associated with improved recovery from disability among stroke survivors [71]. Future studies should explore the factors associated with the lower chances of antidepressant use among those with functional disability. Having three or more chronic diseases was associated with lower likelihood of receiving antidepressant treatment. Multiple factors could explain this finding such as competing demands from other disease conditions [72], risk of adverse events (such as falls) with polypharmacy including antidepressants [73] and drug interactions [74, 75].

In the domain of Personal Health Practices factors, both BMI and smoking status were significantly associated with higher likelihood of receiving antidepressant and combination therapy. The positive association of overweight or obese individuals with depression treatment can be explained by the fact that overweight adults are at an increased risk of depression [76], and that maybe the reason they are reporting higher depression treatment. However, caution should be taken while choosing antidepressants among overweight or obese individuals as some antidepressants (such as SSRIs) have been shown to be associated with abdominal obesity [77]. The present study finding suggests that current smokers had significantly higher likelihood of reporting antidepressants and combination therapy, which is not unexpected as adult smokers with depression smoke heavily [78] and may have higher need to treat depression to reduce smoking. Some antidepressants (such as bupropion) are used for smoking cessation and have been found to be effective in long-term smoking cessation [79], but in the present study population only 15% of current smoker reported using bupropion.

In terms of the external environmental factors, it was surprising to observe that stroke survivors with co-occurring depression who resided in rural areas were more likely to report combination therapy use compared to those residing in metropolitan areas. This finding is inconsistent with existing literature [80, 81], and reason is unclear as there was no significant differences among the study sample residing in metropolitan and rural areas in terms of perceived mental health status, functional disability, and chronic diseases. Future research is warranted to explore the reasons for this difference in metropolitan status. Another interesting finding from this study is that stroke survivors with co-occurring depression residing in Southern region were less likely to report combination therapy use. This is surprising because Southern region is the stroke belt of US and also as per the Darthmouth Atlas of Medicare Prescription Drug use, newer antidepressants use was concentrated in the Southern region [82].

Use of a nationally representative sample of community-dwelling older adults with stroke and co-occurring depression and use of a broad set of individual-level variables including pain, are some of the strengths of this study. However, limitations of this study need to be kept in mind while interpreting the findings. MEPS uses self-reported data that are subject to potential recall bias. However, in order to minimize recall bias, interviews are conducted at regular intervals of 4–5 months by MEPS [20]. Sequence of stroke and depression (whether stroke preceded depression or vice-versa) cannot be obtained from the Medical Conditions file. Severity of stroke and depression is unavailable in MEPS data. It cannot be ascertained from MEPS whether psychotherapy was used specifically for depression or for other conditions (such as anxiety). This is also true for antidepressant use (whether it is specific for depression or it was for other conditions such as anxiety, sleep or appetite stimulation). Inability of establishing cause-effect relationship remains one of the inherent drawbacks of studies utilizing secondary data and hence limitations such as the exact reason for using any pharmacologic or non-pharmacologic option remains elusive. MEPS publically available data provide three digit ICD-9-CM codes that can lead to misclassification bias, however, stroke and depression ICD-9-CM codes used in this study have been validated and used in previous studies utilizing MEPS data. One of the other limitations of this study was that we were not able to segregate stroke subtypes (ischemic and hemorrhagic) as individuals with co-occurring hemorrhagic stroke and depression (*n* = 3) was extremely small. And finally, given the range of years of data (2002–2012) used in this study, it may not represent the current status of depression treatment among stroke survivors in the US.

## Conclusions

Despite the limitations, this is the first study to date to provide national level estimates of depression treatment among community-dwelling older adults with stroke and co-occurring depression. Nearly 90% of the study sample received some form of depression treatment, with SSRIs reported as the most widely used class of antidepressant, and sertraline being the most reported individual antidepressant agent. Use of depression treatment was associated with several individual-level factors such as age, education, poverty status, perceived mental health status, functional disability, presence of other chronic conditions, BMI, smoking, metropolitan status, and region. Future longitudinal studies are warranted to assess the comparative treatment benefits of antidepressants (classes as well as individual antidepressants), psychotherapy and their combination that can add to the current knowledgebase and eventually improve different domains of quality of care (such as effectiveness, safety and patient centeredness). Future studies should also assess the incremental healthcare expenditures among stroke survivors due to the presence of depression and also if depression treatment reduces healthcare expenditure in this vulnerable population.

## Additional file

Additional file 1: Detailed Multum Lexicon codes used to identify sub-classes of antidepressants. Table S1. Multum Lexicon Therapeutic Class Code. (DOCX 12 kb)

## Abbreviations

ADL: Activity of Daily Living
AHRQ: Agency for Healthcare Research and Quality
AOR: Adjusted Odds Ratio
BMI: Body mass index
CI: Confidence Intervals
IADL: Instrumental Activity of Daily
ICD-9-CM: International Classification of Diseases, 9th Edition, Clinical Modification
MAOI: Mono-Amine Oxidase Inhibitors
MEPS: Medical Expenditure Panel Survey
MEPS-HC: Medical Expenditure Panel Survey-Household Component
MEPS-IC: Medical Expenditure Panel Survey-Insurance Component
NDC: National Drug Code
NHIS: National Health interview Survey
OR: Odds Ratio
PPAZ: Phenylpiperazine Antidepressants]
RCT: Randomized Controlled Trial
SNRI: Serotonin–Norepinephrine Reuptake Inhibitor
SSRI: Selective Serotonin Reuptake Inhibitor
TCA: Tricyclic Antidepressants
US: United States
WHI: Women’s Health Initiative
