# Supplementary material for: Prevalence, patterns and predictors of depression treatment among community-dwelling older adults with stroke in the United States: a cross sectional study
Source: BMC Psychiatry. 2018 May 16;18:130. doi: 10.1186/s12888-018-1723-x (PMC5956759; doi:10.1186/s12888-018-1723-x)
Supplement: Supplementary file 1 — Detailed Multum Lexicon codes used to identify sub-classes of antidepressants. Table S1. Multum Lexicon Therapeutic Class Code. (DOCX 12 kb) [file 12888_2018_1723_MOESM1_ESM.docx]

Supplementary tables: Detailed Multum Lexicon codes used to identify sub-classes of antidepressants

| **Table S1: Multum Lexicon Therapeutic Class Code** | | | | | |
| --- | --- | --- | --- | --- | --- |
| **Level 1** | | **Level 2** | | **Level 3** | |
| **CAT ID** | **Category Name** | **CAT ID** | **Category Name** | **CAT ID** | **Category Name** |
| 242 | Psychotherapeutic Agents | 249 | Antidepressants | 76 | Miscellaneous Antidepressants |
| 242 | Psychotherapeutic Agents | 249 | Antidepressants | 208 | SSRI Antidepressants |
| 242 | Psychotherapeutic Agents | 249 | Antidepressants | 209 | Tricyclic Antidepressants |
| 242 | Psychotherapeutic Agents | 249 | Antidepressants | 250 | MAOI |
| 242 | Psychotherapeutic Agents | 249 | Antidepressants | 306 | Phenylpiperazine Antidepressants |
| 242 | Psychotherapeutic Agents | 249 | Antidepressants | 307 | Tetracyclic Antidepressants |
| 242 | Psychotherapeutic Agents | 249 | Antidepressants | 308 | SNRI antidepressants |

*Abbreviations* --- CAT ID: Category ID; SSRI: Selective Serotonin Reuptake Inhibitor; SNRI: Serotonin–Norepinephrine Reuptake Inhibitor; MAOI: Monoamine Oxidase Inhibitors.
